# Method for Recovery and Immunoaffinity Enrichment of Membrane Proteins Illustrated with Metastatic Ovarian Cancer Tissues

**DOI:** 10.1155/2012/838630

**Published:** 2012-07-12

**Authors:** Luke V. Schneider, Varsha Likhte, William H. Wright, Frances Chu, Emma Cambron, Anne Baldwin-Burnett, Jessica Krakow, Gary B. Smejkal

**Affiliations:** ^1^Target Discovery, Inc., 4030 Fabian Way, Palo Alto, CA 94030, USA; ^2^Pressure Biosciences, Inc., 14 Norfolk Avenue, South Easton, MA 02375, USA; ^3^Hubbard Center for Genome Studies, University of New Hampshire, Durham, NH 03824, USA

## Abstract

Integral membrane proteins play key biological roles in cell signaling, transport, and pathogen invasion. However, quantitative clinical assays for this critical class of proteins remain elusive and are generally limited to serum-soluble extracellular fragments. Furthermore, classic proteomic approaches to membrane protein analysis typically involve proteolytic digestion of the soluble pieces, resulting in separation of intra- and extracellular segments and significant informational loss. In this paper, we describe the development of a new method for the quantitative extraction of intact integral membrane proteins (including GPCRs) from solid metastatic ovarian tumors using pressure cycling technology in combination with a new (ProteoSolve-TD) buffer system. This new extraction buffer is compatible with immunoaffinity methods (e.g., ELISA and immunoaffinity chromatography), as well as conventional proteomic techniques (e.g., 2D gels, western blots). We demonstrate near quantitative recovery of membrane proteins EDG2, EDG4, FASLG, KDR, and LAMP-3 by western blots. We have also adapted commercial ELISAs for serum-soluble membrane protein fragments (e.g., sVEGFR2) to measure the tissue titers of their transmembrane progenitors. Finally, we demonstrate the compatibility of the new buffers with immunoaffinity enrichment/mass spectrometric characterization of tissue proteins.

## 1. Introduction

Integral membrane proteins, particularly G-protein-coupled receptors (GPCRs), are the biological targets for half of all the small molecule pharmaceuticals on the market today [1–3]. Membrane transport proteins, such as P-glycoprotein and related efflux pumps, are thought to impart chemotherapy agent resistance by transporting the drugs from the cytoplasm faster than they can diffuse back, thus lowering the effective drug concentrations at the site of action [4]. Even the common cold (rhinovirus) invades the cell by first binding to specific cell surface proteins [5–7], at least some of which are thought to involve glycosylated and sialylated extracelluar domain recognition sites [7, 8]. Clearly, integral membrane proteins play key biological roles in cell signaling, transport, and pathogen invasion. As such, membrane proteins also play key clinical roles in drug efficacy and resistance and should have a larger role in clinical diagnostics and personalized medicine. However, quantitative clinical assays (e.g., immunosorbent assays) for this important class of proteins remain elusive and are generally limited to serum-soluble extracellular fragments. Many serum markers for cancer detection and treatment monitoring—such as CA-125 (a serum-soluble fragment of mucin-16 approved for recurrence monitoring of ovarian cancer), CA 15-3 (a serum-soluble fragment of mucin-1 approved for recurrence monitoring of breast cancer), sVEGFR (a serum-soluble fragment of the vascular endothelial growth factor receptor that is implicated as a prognostic marker in lung cancer) [9], and sEGFR (a serum-soluble fragment of endothelial growth factor receptor that is implicated as a theranostic marker for trastuzumab treatment in breast cancer) [10]—are currently only accessible for clinical assays once extracellular fragments are shed from the tumor cell membranes into the circulatory system. Other membrane protein biomarkers—such as HER-2/neu (an oncogenic growth factor receptor approved for use in herceptin therapy guidance) [11] and the estrogen receptor (an indicator for hormonal therapy in breast cancer) [12]—are currently only accessible through gene-based assays. Yet, genetic assays are unable to detect potentially clinically relevant posttranslational modifications, such as glycosylation, phosphorylation, acetylation, ubiquitination, and editing. Furthermore, as has been well established for more than a decade, measurements of mRNA levels, which are produced transiently, do not correlate well to protein levels, which accumulate over time [13, 14].

### 1.1. Membrane Protein Recovery and Purification

Classically, detergents are used to extract membrane proteins from biological membranes. Detergents also mediate membrane protein solubility in aqueous solutions, which is a prerequisite for further protein purification [15]. The surfactant concentrations required to keep most membrane proteins in aqueous solution also typically denature immunoglobulins, precluding their use for immunoaffinity purification and enrichment. Therefore, purification of membrane proteins is often very tedious and is made more so because surfactants can only partially mimic the lipid bilayer environment of the protein in nature [16]. Thus, many membrane proteins no longer retain their native biological conformations or activities in surfactant solutions [17], except in isolated cases [18]. Furthermore, not all proteins can be recovered efficiently with the same surfactant. Mitic et al. showed how the recovery of claudin-4 (with four transmembrane sequences) from insect cell cultures failed to consistently track total protein recovery over 37 different surfactants tested, ranging from 0 to 169% of the sodium dodecyl sulfate (SDS) control [19]. Surfactants also create limitations on further proteomic analysis of membrane proteins, since subsequent polyacrylamide gel electrophoresis of the recovered proteins generally requires SDS, or other ionic surfactants such as perfluorooctanoic acid [20]. With the exception of newer acid-cleavable forms [21], surfactants can produce ionization problems for mass spectrometric analyses, except at very low concentrations [22, 23], which are too low to support solubility of membrane proteins. Surfactants also bind to surfaces, significantly altering the behavior of liquid chromatographic media [24].

Because of the problems surfactants pose in chromatographic and mass spectrometric proteomic analyses, several membrane protein extraction schemes have been reported based on chaotropic agents and organic solvents. Jacks et al. report a 4 : 1 : 1 mixture of ethanol : acetonitrile : water as being useful for recovering membrane proteins of mitochondrial and spherosomal origin in a system that is optically transparent in the range of 200–700 nm [25]. The lower end of this optical range is particularly useful for studying protein structure by circular dichroism or quantification by absorbance. Zhang et al. report on the use of aqueous methanol, trifluoroethanol, and aqueous urea for the extraction of membrane proteins from red blood cells, finding that each solvent system liberated different membrane proteins [26]. Cordwell has advocated the use of a series of potent chaotropic agents and detergents (increasing progressively in strength) for protein extraction and fractionation, ending with thiourea and amidosulfobetaine-14 [27]. He applied this method to Gram-negative bacteria, cultured mammalian cells, and heart tissue. 

In all these cases, the more readily soluble proteins are generally recovered first from the patient sample in standard aqueous buffers from which they can be more readily purified and assayed since all the solvents, detergents, and chaotropic agents necessary to recover and solubilize the integral membrane proteins are incompatible with the downstream separation and purification schemes. Therefore, the only applicable immunoaffinity technique that can be used on most membrane proteins after surfactant extraction is western blotting [28], which has only been sparing applied in clinical settings (e.g., early human immunodeficiency virus testing [29], early bovine spongiform encephalopathy testing [30], and Lyme disease [31]). Even these were quickly replaced when more robust nucleic acid or enzyme-linked immunosorbent assay (ELISA) alternatives became available.

### 1.2. Proteolysis of Intra- and Extracellular Domains

 Another classic proteomic approach to membrane protein analysis involves digestion of the generally soluble intra- and extracellular domains from the generally insoluble transmembrane regions. Nühse et al. used this approach in combination with immobilized metal ion affinity chromatography (IMAC) to study the phosphopeptides resulting from trypsin treatment of the plasma membrane fraction isolated from cultured Arabidopsis cells [32]. However, membrane proteins can be refractory to digestion, particularly to trypsin, and nonspecific digestion enzymes (e.g., pepsin and elastase) are more commonly employed for this purpose [33]. The challenge, therefore, is to control the degree of digestion so that optimal recoveries are obtained. The resulting mixtures of partially digested peptides limit detection of the subsequent peptides by distributing the available signal over more distinct, but related, mass spectrometric species. Furthermore, this approach is generally only suited for global proteomic analysis by liquid chromatography and mass spectrometry since the resultant peptides are often not immunologically active and mixtures of peptides are created from all the membrane proteins found in the sample (both diagnostic and nondiagnostic).

With the exception of qualitative immunohistochemistry, clinical exploitation of integral membrane proteins has heretofore been limited by our ability to recover these proteins in a form suitable for quantitative immunoassays and rapid proteomic characterization. In this paper, we describe a new approach for the combined quantitative recovery of both cytosolic and integral membrane proteins in a buffer system immediately suitable for direct use in immunosorbent assays and subsequent mass spectrometric analyses. This approach uses the commercial ProteoSolve-TD1 and -TD2 extraction buffers, developed in our laboratory and commercialized by Pressure Biosciences (PBI), in combination with PBI's pressure cycling technology (PCT). This new approach is demonstrated by extraction and analysis of several different biomarker proteins from fresh frozen human metastatic ovarian tumor tissues.

## 2. Material and Methods

### 2.1. Tissue Preparation and Protein Extraction

Fresh frozen samples of metastatic ovarian tumors (surgically isolated from the omentum of several different individuals) were purchased from Bio-Options (Fullerton, CA). All samples were reported to have been obtained from surgical resections of stages III and IV ovarian cancer patients. The frozen tumor samples were finely diced and mixed prior to extraction to provide better sample homogeneity.

### 2.2. Cryogenic Grinding

Diced tumor tissue (200 mg) was weighed into an aluminum weigh boat, precooled over dry ice, to keep the samples frozen during processing. A mortar and pestle were precooled by addition of liquid N_2_ until a permanent lake of liquid N_2_ could be maintained in the mortar. The tissue sample was added to the lake of liquid N_2_ and cryogenically ground under liquid N_2_ to a fine powder (about the consistency of corn starch). Additional liquid N_2_ was added as necessary. After grinding, the liquid N_2_ was allowed to evaporate, and the frozen tissue along with any frozen condensate was transferred to a PULSE tube (an integral part of the Barocycler device), which was precooled in a bed of dry ice.

### 2.3. Extraction Buffers

Three different buffer systems were used to extract proteins from the ground tumor tissue samples. The control extraction buffer, adapted from Song et al. for the homogenization of liver tissue for subsequent protein recovery and analysis, consisted of 20 mM HEPES adjusted to pH 7.5 with NaOH [34]. This buffer also forms the basis of the ProteoSolve-TD1 extraction buffer (Pressure Biosciences, South Easton, MA), which contains additional agents for membrane protein extraction and stabilization. The ProteoSolve-TD2 extraction buffer (Pressure Biosciences) was subsequently formulated with additional agents to stabilize the ground tissue dispersion during Barocycler operation, improving the reproducibility of protein extraction between samples. All these buffers were formulated to be compatible with subsequent protein labeling chemistries (e.g., aldehyde Schiff-base, N-hydroxysuccinimide, or iodoacetamide) to facilitate quantitative stable isotope work, such as isotope-coded affinity tags (ICATs) [35], isobaric tags for relative and absolute quantitation (iTRAQ) [36], and mass defect (isotope-differentiated binding energy shift tags, IDBEST) [37].

### 2.4. Barocycler Extraction

The NEP2320 Barocycler (Pressure Biosciences) was precooled with a circulating water bath to 4°C prior to use. All extraction buffers were refrigerated (0–4°C) overnight prior to use and used cold. Commercial protease and phosphatase inhibitor cocktails (P8340, P5726, and P2850, Sigma Aldrich, St. Louis, MO) were added to each buffer according to package directions immediately prior to use. PULSE tubes were loaded according to the manufacturer's instructions using 1.3 mL of the appropriate buffer in each tube. The tubes were immediately processed in the Barocycler (20 cycles from 0 to 35,000 psi for 20 sec on each cycle).

The resulting extracts were viscous and were treated with 25 *μ*L of micrococcal nuclease from *Staphylococcus aureus* (New England Biolabs, Ipswich, MA) reconstituted at 1,000 units per mL per the manufacturer's instructions, for 15 min on ice. The resulting extract was recovered with a transfer pipette and placed in a 2 mL microfuge tube along with any residual pellet. The pellets with HEPES and TD1 extraction buffers appeared as soft sticky disks generally pressed against the center frit of the PULSE tube. Significantly less pellet was formed in the TD2 extraction buffer, and the TD2 postextraction pellet proved to be very friable.

Insoluble materials were recovered from each extract by centrifugation (13,000 ×g for 15 minutes at 4°C). The clarified extracts were recovered by aspiration to a second microfuge tube. The pellets were resuspended in 1 mL of TD1 buffer by passing them through a 21-gauge syringe needle several times to disperse the pellet. Because the pellets were not washed, they contain some residual soluble protein. Both the pellets and clarified extracts were stored in aliquots at −80°C prior to use.

### 2.5. 2-D Gel Electrophoresis

Aliquots (45 *μ*L) of both the HEPES- and TD1-clarified extracts were diluted into 365 *μ*L of ProteoSolve-IEF buffer (Pressure BioSciences, Inc.). First dimension separation was carried out with 200 *μ*L of each diluted extract on the computer-controlled IsoelectrIQ^2^ IEF apparatus (Proteome Systems, Ltd. Sydney, AU) using 11 cm, pH 3–10 ReadyStrip IPG strips (Bio-Rad Laboratories, Inc., Hercules, CA). Separation was programmed with current limited to 50 *μ*A/strip in two steps: twelve hours on a concave voltage ramp set to start at 100 V and end at 10,000 V, followed by a constant voltage for 8 hours at 10,000 V. The strips were removed at 90 kV-h. The second dimension was performed on Criterion 8–16% polyacrylamide Tris-HCl precast gels in a Dodeca Cell (both from Bio-Rad Laboratories), equipped with the Thermo-EC 570–90 power supply at constant current of 60 mA/gel for 2 h. Gels were fixed and stained with a ProteomIQ Blue Colloidal Coomassie gel stain as described previously [38]. Gel images were acquired on a UMAX PowerLook III flatbed scanner as 48-bit color TIFF files and converted to 16-bit grayscale using ImageJ software (NIH). Image analysis was performed using Ludesi REDFIN 3 software (Ludesi AB, Malmö, Sweden).

### 2.6. Western Blots

Aliquots (17.5 *μ*L) of each clarified extract and corresponding pellet suspension were denatured by the addition of 5 *μ*L of 4x NuPAGE LDS Sample Buffer (Invitrogen), 2 *μ*L of 1 M dithiothreitol, followed by heating to 90°C for 10 minutes. The samples were centrifuged at 13,000 ×g (5 min) and the entire contents run on precast 4–12% Bis-Tris NuPAGE minigels, using the XCell SureLock system (Invitrogen, Carlsbad, CA), according to the manufacturer's instructions. Both SeeBlue and Magimark (Invitrogen) were used as molecular weight markers on each gel according to package instructions. Proteins were transferred to PVDF membranes at 65 V for 2 hours using a transfer buffer consisting of 20 mM TRIS, 160 mM glycine, and 0.04% SDS.

The PVDF membranes were blocked on a rotary shaker at room temperature with two different blocking buffers. A blocking buffer consisting of 100 mM phosphate buffered saline with 0.05% Tween, 0.01% Thimerosal, and 10% nonfat milk was used for the FASLG, NRP1, KDR, LAMP-3, BCL-2, CCNE2, and AKT blots. These were incubated for 10 min before primary antibody addition. The blocking buffer used for the EDG4, EDG2, GPC3, and TUBB blots consisted of 25 mM Tris, 0.15 M NaCl, 0.1% Tween-20, and 0.01% thimerosal at pH 7.4 containing 2% nonfat milk. The second set of membranes were blocked for 1 h before addition of the primary antibody. In both cases, the blocking buffer used for incubation was removed before addition of fresh buffer with the primary antibody. Primary antibodies were added at a nominal concentration of 1 *μ*g/mL in 10 mL of the respective blocking buffers for FASLG, NRP1, KDR, LAMP-3, BCL-2, CCNE2, and AKT blots, and a nominal concentration of 0.5 *μ*g/mL in 12 mL for the EDG4, EDG2, GPC3, and TUBB blots (except with 1% nonfat milk). Each blot was incubated with the primary antibody overnight on a rotary shaker at 0–4°C. Primary antibodies consisted of affinity-purified polyclonal antibodies against each biomarker purchased from various sources (Table 1). Appropriate cell lysates were used as positive controls for each of the antibodies in the western blots (Table 1).

After overnight incubation with the primary antibody, the blots were washed 4-5 times with their respective blocking buffers (without the nonfat milk). Washed blots were placed in 4 mL of the respective blocking buffer (without the nonfat milk) to which the appropriate antiprimary, secondary HRP-conjugated antibody (Cell Signaling) was added as supplied at a 1 : 1000 dilution for the FASLG, NRP1, KDR, LAMP-3, BCL-2, CCNE2, and AKT blots and 1 : 10,000 dilution for the EDG4, EDG2, GPC3, and TUBB blots. Blots were incubated with the HRP-conjugated secondary antibodies for 1 h at room temperature on a rotary shaker. Blots were again washed as described above and developed using Supersignal West Femto substrate (Pierce, Thermo-Fisher) following the manufacturer's recommendations. The chemiluminescent images were collected using a Fluorchem SP gel imager (Alpha Innotech, San Leandro, CA). The grey scale was inverted during postprocessing.

### 2.7. Sample Preparation for Immunoaffinity Techniques

Ovarian tumor samples extracted with TD2 buffer using the Barocycler (as described above) were diluted 1 : 10 in ProteoSolve-TDilute (Pressure Biosciences) containing both phosphatase and protease inhibitors (previously described) prior to any immunoaffinity work.

### 2.8. Enzyme-Linked Immunosorbent Assays

The effect of the diluted TD2 buffer on immunoassays was evaluated in several commercial ELISA kits, including human transferrin kit (Bethyl Laboratories, Montgomery, TX), Quantikine MMP-2 and MMP-3 (R&D Systems, Minneapolis, MN), and PathScan total p53 and PathScan total AKT1 (Cell Signaling, Danvers, MA). Immunoassays were performed following the manufacturer's instructions and in parallel with standards reconstituted in the diluted TD2 buffer (described above). Rate assays (change in absorbance with time) were performed, instead of the standard single time point, to ascertain any residual effects of the TD2 buffer components on the amplification step of the assay (i.e., modulation of horseradish peroxidase [HRP] enzyme kinetics or quenching of substrate color development).

Nonlinear least squares curve fit of the antibody binding (1) to the standards prepared in each buffer system was used to get the apparent affinity constants (*K*_*aff*⁡_). While the total antigen concentration [An] is known in the standard curve, the total antibody concentration [Ab] and the affinity constant were determined simultaneously by nonlinear curve fit. The one sigma error of the estimate in each fitted parameter was determined from the Jacobian matrix.

(1) Rate=k1+kaff⁡[An]−kaff⁡[Ab]2kaff⁡+4kaff⁡2[An][Ab]+(kaff⁡[An]−kaff⁡[Ab]−1)22kaff⁡.

The ovarian TD2 extracts (after 1 : 10 dilution in ProteoSolve-TDilute) were also run in each assay to determine the effective biomarker concentrations in the extract. Because of this dilution, the highest tissue concentration tested was 15 mg of tissue/mL, which in a 100 *μ*L ELISA sample well is the equivalent of 1.5 mg of tissue.

### 2.9. Immunoaffinity Enrichment of Specific Biomarkers

Antibody-conjugated PhyTips (PhyNexus, Sunnyvale, CA) were used for all immunoaffinity enrichment experiments. The experiments were conducted on a PhyNexus MEA robot system (PhyNexus, Sunnyvale, CA) using deep well plates. Each tip contained 100 *μ*L fluidized beds of AminoLink Resin (Pierce Protein Research, Thermo-Fisher Scientific, Rockford, IL) conjugated to affinity-purified polyclonal antitransferrin antibody (Bethyl Laboratories, Montgomery, TX) and packed in glycerol. The antibody conjugation procedure is described below. The sample consisted of 1 mL of ovarian tumor extract prepared in TD2 buffer and diluted (1 : 10 in ProteoSolve-TDilute) as described above. Nonspecific goat IgG (Equitech-Bio, Kerrville, TX) was added to the sample (9 mg per 10 mL of diluted sample) to suppress histone binding to the antibodies present on the bead surface [39].

Glycerol (used to pack and store the PhyTips) was found to significantly alter flow through the PhyTips and suppress ionization in the subsequent mass spectrometric analyses. This glycerol was removed by washing the PhyTips with 100 mM PBS (pH 7.2) in two stages using 96-well deep well trays (Seahorse Labware, no. S30009, 2 mL per well). The first stage wash consisted of four successive quick rinses in single draw and expel cycle each of 1 mL at a 2 mL/min flow rate (with 2 min holds at the top and bottom of each cycle). This removed the glycerol surrounding the bead bed, but left glycerol saturating the beads themselves. Diffusion of the glycerol out of the pores of the beads in the second stage required four additional washes consisting of a 0.5 mL draw followed by 60 cycles of 0.3 mL volume at a 2 mL/min flow rate (with 2 and 10 sec holds at the bottom and top of each cycle). This totaled about 30 min in each 20 mL wash volume (for 12 tips). Immediately following glycerol removal, the tips were immersed in a trough of an 8-row deep trough trays (Seahorse Labware, no. S30020, 32 mL per trough) containing 10 mL of diluted tumor sample. Next, 0.5 mL of sample was drawn into each tip, and 0.3 mL was cycled 240 times at 2 mL/min (with 2 and 10 sec holds at the bottom and top). This totaled about 2-hour for sample binding. Sample binding was immediately followed by a stringency wash in 4.17 M NaCl with 83 mM NaPO_4_ (pH 7.2) for 60 × 0.5 mL cycles at 2 mL/min (with 2 and 10 sec holds at bottom and top). This was followed by buffer exchange into 150 mM pyridinium acetate (pH 6). Five washes in 10 mL of pyridinium acetate in a trough (0.5 mL with 48 cycles of 0.3 mL each at 2 mL/min with 2 and 10 sec holds at the bottom and top of each cycle) were required to diffuse all the salts from within the bead pores. Antigens were subsequently eluted directly into 2 mL microfuge tubes containing 0.6 mL of 50% aqueous acetic acid (0.5 mL draw with 30 cycles of 0.3 mL at 2 mL/min). The eluates were dried overnight in a SpeedVac (Savant). The residual pyridium acetate is a volatile buffer, which evaporated with the water in the SpeedVac.

### 2.10. Antitransferrin Antibody Conjugation to PhyTips

Custom PhyTipswere purchased from PhyNexus (Sunnyvale, CA). These were1.1 mL volume pipette tips packed by PhyNexus with 100 **μ**L of AminoLink beads (ThermoFisher) in a fluidized bed configuration. Affinity-purified, carrier-free, goat polyclonal antitransferrin antibodies (no. A80-128A, Bethyl Laboratories, Montgomery, TX) were dissolved at 83 *μ*g/mL in 100 mM PBS (pH 7.8) with 33 mM sodium cyanoborohydride (NaCNBH_3_). A 0.6 mL quantity of the antibody solution was placed in the well of a deep-well plate for each tip. A 0.5 mL quantity of the antibody solution was drawn into each glycerol-free PhyTips (washed as described above) and processed for 960 cycles of 0.3 mL at 2 mL/min (6 h). Unreacted AminoLink aldehydes were then quenched with a 0.5 mL draw and 60 cycles of 0.3 mL each at 2 mL/min (0.5 h) in 1 M tris(hydroxymethyl)-aminomethane chloride (pH 7.8) with 33 mM NaCNBH_3_. Residual TRIS and cyanoborohydride were removed with five washes (0.5 mL draw and 60 cycles of 0.3 mL each) in 100 mM PBS, the last of which contained 0.05% sodium azide. The tips were then packed with glycerol and stored refrigerated. An average of 35 *μ*g Ab was bound to each PhyTip, as determined by UV_280_ absorbance change in the conjugation solution.

### 2.11. MALDI-MS Analysis

Immunoaffinity-enriched transferrin from ovarian tumor extracts was identified by peptide mass fingerprinting. The dried eluates (described above) were dissolved in 0.1 mL of 20 mM ammonium bicarbonate (pH 8.2), simultaneously reduced and capped by the addition of 2 *μ*L each of 2-vinylpyridine (50 mM in isopropanol) and triethylphosphine (25 mM in isopropanol) at 37°C for 1 h, following the procedure described by Hale et al. [40]. After capping, the eluates were digested by adding 2.5 *μ*L of trypsin (Sequencing Grade-Modified, Promega, Madison, WI), reconstituted at 100 *μ*g/mL in 20 mM ammonium bicarbonate, for 2 h at 37°C. Addition of 0.11 mL of HPLC grade acetonitrile quenched the digestion, and the digest was evaporated overnight in a SpeedVac. The pellet was resuspended in 25 *μ*L of MALDI matrix (*α*-cyano-4-hydroxycinnamic acid dissolved at 5 mg/mL in 50 : 50 acetonitrile : water with 0.1% trifluoroacetic acid), and 1 *μ*L of the digest was spotted on a stainless steel MALDI plate and analyzed using a Q-TOF Premier (Waters, Milford, MA). The resulting monoisotopic peptide peaks were selected using mMass [41], and matching proteins were identified using MASCOT to search the Swiss-Prot protein database [42].

## 3. Results and Discussion

### 3.1. Global Protein Recovery

**Figure 1:**
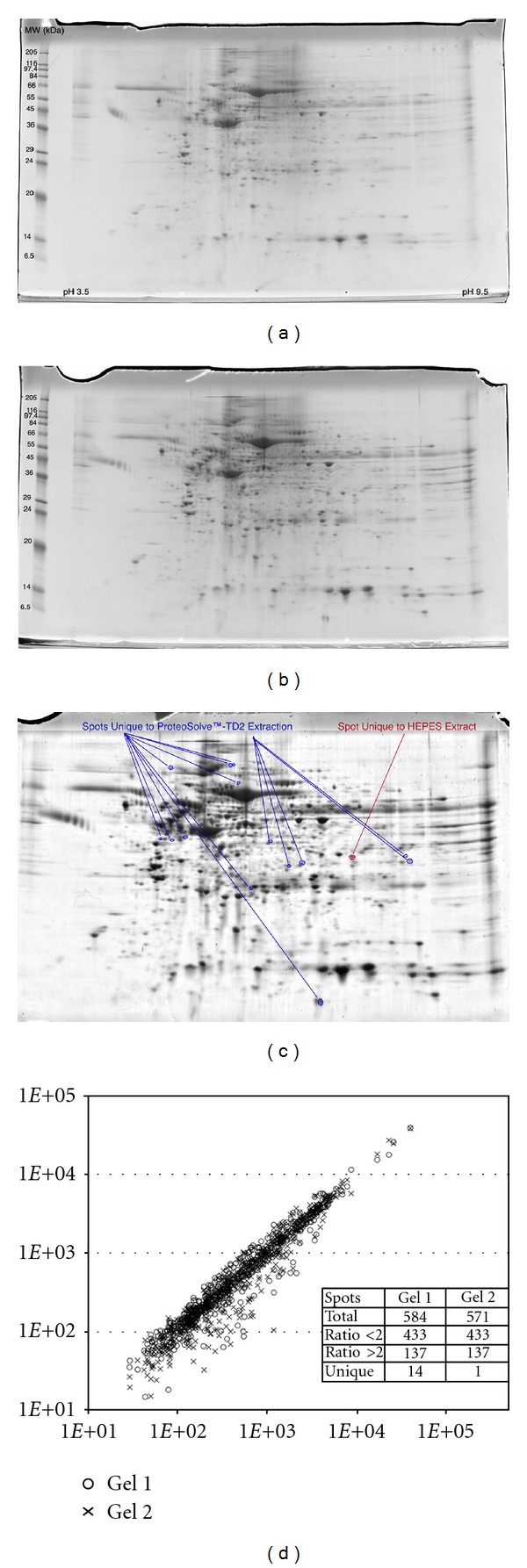
2D gel analysis of proteins extracted from the same metastatic ovarian tumor sample by (a) HEPES buffer (Gel 1) and (b) TD2 buffer (Gel 2). Each extraction was conducted for 30 pressure cycles (30,000 psi for 20 sec followed by 0 psi for 20 sec) at a concentration of 75 mg tissue/mL of extraction buffer using the Barocycler. The samples were subsequently diluted to 8.6 mg/mL in ProteoSolve-IEF (a denaturing IEF gel buffer) for gel analysis. Automated gel image analysis of the Coomassie-stained gels suggests that 97% of the protein spots are shared between the two gels in position with most (74%) of the same abundance (d). One spot is uniquely found in the HEPES control, and 14 spots are unique to the ProteoSolve-TD2 extraction.

In order to show equivalence to classic extraction buffers, we performed a global proteomic analysis (2D gel electrophoresis) using separate 100 mg aliquots of a cryogenically ground metastatic ovarian tumor sample pool (sourced from multiple patients). Using the Barocycler, the first aliquot was extracted in HEPES buffer and a second processed in the TD2 buffer. The clarified extracts were diluted to 8.6 mg of tissue/mL in denaturing IEF buffer for 2D gel analysis. Comparison of the resulting gels by image analysis (Figure 1) reveals few differences in the more abundant protein species recovered. Of 585 discrete protein spots identified, 97% were common in both position and abundance between the two gels. Only 14 protein spots were unique to the TD2 extraction buffer gel. One spot was unique to the HEPES extraction buffer. These 15 differences were all in less abundant proteins. Therefore, TD2 buffer appears fully compatible with classic gel electrophoretic methods with little alteration in recovery of the more abundant proteins.

### 3.2. Recovery of Specific Proteins

Only the most abundant proteins can be seen in Coomassie-stained gels. Thus, the above analysis tells us little about the quantitative extraction of membrane proteins. We, therefore, selected a number of representative biomarkers from different protein classes for more detailed analysis by western blots. In particular, we were interested in determining how much protein of each class was left behind unrecovered in the insoluble pellets. To this end, the insoluble Barocycler pellets from each condition tested were recovered and treated by boiling in SDS-PAGE sample buffer. These SDS extracts of the pellets were run side by side in western blots with the clarified extracts at similar “effective” tissue concentrations.

**Figure 2:**
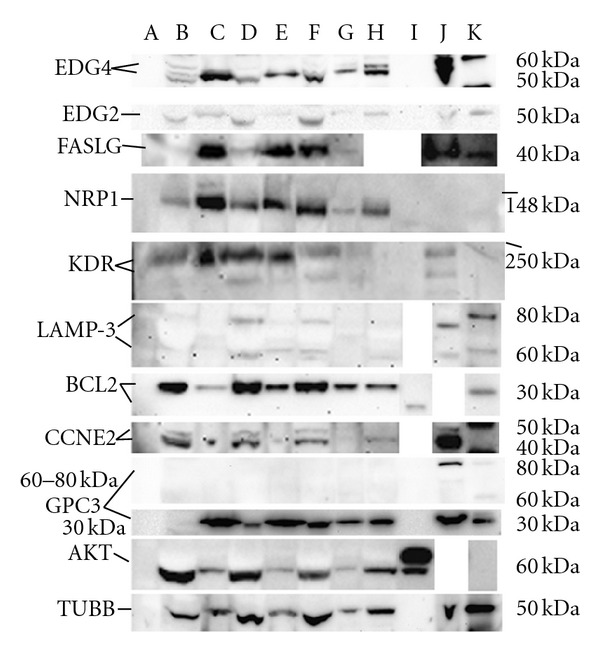
A series of western blots for various membrane, nuclear, and cytosolic proteins (Table 2) extracted from metastatic ovarian tumor samples. The extracts were prepared using 3 different extraction buffers (lane B = HEPES buffer extract, lane D = TD1 buffer extract, and lane F = TD2 buffer extract). Lane H is a duplicate of lane F at 1/3 the protein loading. The corresponding insoluble proteins trapped in the pellets recovered after each extraction are also shown in the adjacent lanes (lane C = HEPES pellet, lane E = TD1 pellet, and lane G = TD2 pellet). Either a purified recombinant protein control (lane I) or a human cell line extract control (lane J) was used in each blot (both were used for EDG4, NRP1, and KDR). Lanes A (see blue, Invitrogen) and K (Magic Mark, Invitrogen) are molecular weight markers. Lane A is only visualized in visible light, not in the chemiluminescent images shown. Both of these markers (see blue from a white light image not shown) were used to determine the molecular weights shown (on the right hand side). Gaps between lanes are provided only for alignment purposes, and the lanes are from the same gels.

Tissue extractions were performed with HEPES, TD1, and TD2 buffers at 150 mg tissue/mL buffer concentrations. The Barocycler extracts were centrifuged to recover an insoluble pellet and a soluble protein extract as separate samples. The extracts were diluted directly into 4x LDS-PAGE sample buffer (Invitrogen, Carlsbad, CA) to an equivalent gel loading concentration of 110 mg tissue/mL. All the pellets were resuspended in TDilute buffer to an equivalent concentration of 200 mg of tissue/mL to create a fine suspension. An aliquot of this suspension was diluted in LDS-PAGE sample buffer to an equivalent gel loading concentration of 140 mg tissue/mL. A series of western blots (Figure 2) were prepared from these extracts and pellets. Each blot was probed for a different protein.

Each extract and corresponding pellet sample were obtained from the same PULSE tube (i.e., the same tissue preparation). Therefore, it is possible to determine the relative abundance of each protein seen between the extract and pellet for each buffer. However, different PULSE tubes are used for each of the different extraction buffers tested. Because of water condensation during the weighing of frozen tissues, the amounts of tissue may vary between PULSE tubes. This makes direct cross-comparison of absolute protein recovery between buffers impractical. However, recovery determinations between the clarified extract and its corresponding pellet are possible.

Detailed descriptions of the specific proteins analyzed by western blots (Figure 2) can be found in the Supplementary Materials available online at doi:10.1155/2012/838630. The salient features of these proteins are summarized in Table 2. Key among these features are the number of transmembrane sequences, the theoretical (sequence MW) of the protein and any splice variants, and the reported measured MWs of the protein, including any posttranslational modifications. Alternate gene names are also provided to facilitate searches.

EDG2 and EDG4 are both G-protein-coupled receptors. Two strong overlapping EDG4 bands appear in the gel with nearly equal intensity and differing by less than 2 kDa in weight in the cell line control (lane J) between 50 and 60 kDa. Single strong EDG4 bands are seen in all the ovarian tumor samples at 55 kDa, except the HEPES extract (lane B). Three weak bands are seen in the HEPES extract at 55, 56, and 57 kDa, only one of which may correlate to EDG4. Clearly, little EDG4 is extracted into the HEPES buffer in the Barocycler since the amount extracted from the HEPES pellet (lane C) is in great excess to any of the bands seen in the HEPES extract. It is also possible that all the bands seen in this extract may be cross-reactive protein species because the band pattern is so different from that observed in any of the other samples. The strongest EDG4 band in both the TD1 and TD2 extracts (lanes D and F, resp.) appears at a slightly lower apparent molecular weight than the EDG4 band in the corresponding pellets. We note, however, that the molecular weights for the EDG4 bands observed in the 1/3 dilution of the TD2 extract (lane H) appear at the higher molecular weight observed for both the cell line control and the dominant EDG4 band observed in the TD1 and TD2 pellets. Therefore, we suspect that the EDG4 protein in both the TD1 and TD2 extracts is running at a slightly lower molecular weight, either because the protein load in these lanes is too high or the SDS fails to fully displace bound membrane lipids found in the insoluble pellet fractions. An additional weak band is seen at 56-57 kDa in the 1/3 dilution of the TD2 pellet (lane H), but in none of the other samples, and may be an artifact.

An EDG2 band is seen in all samples at or just below 50 kDa, including the cell line control (lane H). The protein appears to run at a slightly higher molecular weight when recovered with hot SDS from the Barocycler pellets than when isolated from the Barocycler extracts. This may reflect incomplete displacement of adsorbed lipids by SDS from the insoluble protein found in the pellets. However, it may also merely reflect differences in protein concentrations between the gel lanes since the 1/3 concentration sample of the TD2 Barocycler extract (lane H) runs closer to the higher-molecular-weight band and appears less distorted. The EDG2 band in the cell line control (lane J) is similarly distorted as the other extracts. EDG2 recovery seems to improve dramatically from the HEPES to TD buffers. Comparison of the TD2 and TD1 buffers in this sample shows only marginal recovery improvement.

Little or no FASLG appears to be recovered in either the HEPES or TD1 extracts from the Barocycler (lanes B and D in Figure 2). FASLG is seen in high abundance in the insoluble pellets from both of these extracts. However, nearly complete recovery of FASLG is seen in the soluble TD2 extract with little remaining in the insoluble pellet. Other experiments (data not shown) suggested that FASLG recovery was variable with TD1 buffer, but was consistently high in the TD2 buffer. FASLG was not recovered in the extract after several attempts with the HEPES buffer.

No evidence of the 72 kDa soluble form of NRP1 is seen on the western blots. The 140 kDa membrane bound form is present in all extracts (lanes B, D, and F). However, NRP1 recovery into the soluble fraction was lowest in HEPES buffer with most of the protein found in the HEPES pellet. About half of the NRP1 appeared to be recovered in the TD1 extract in this experiment. However, NRP1 recovery in the TD1 buffer was inconsistent between trials (data not shown). Almost complete recovery is seen in the TD2 extract with only a trace of NRP1 left in the pellet. This result was consistent between trials (data not shown). NRP1 was not seen in the cell line control. It is possible that the antibody used in these blots was reactive to a variant of the NRP1 protein that was not present in the cell line control used and which presents an epitope that is removed in the creation of the 72 kDa soluble form.

Both the intermediate and mature KDR proteins are apparent in the western blot for both the cell line control and the tumor samples at 200 kDa and 230 kDa, respectively. Partial recovery of the more abundant mature KDR protein is seen in HEPES extract (lane B) with most of the protein left in the HEPES pellet (lane C). The intermediate glycosylated form was not seen in the HEPES extract or pellet, possibly due to its lower solubility in the HEPES buffer than either the TD1 or TD2 buffers. The aqueous solubility of the mature form is expected to be greater due to the higher level of glycosylation. Most of the intermediate glycosylated form appears to be extracted into both the TD1 and TD2 buffers (lanes D and F) with apparently little left in either pellet (lanes E and G). At least some of the more abundant mature form (230 kDa) was seen in all the extracts (lanes B, D, and F, Figure 2) and both the HEPES and TD1 pellets (lanes C and E, Figure 2). The best relative KDR extraction was observed with the TD2 buffer (lanes F and G, Figure 2).

Two bands at 58 kDa and 73 kDa are seen for the LAMP-3 protein in the western blot (Figure 2). It is not clear if the upper band is a cross-reactive antigen or a hyperglycosylated version of the protein, but it is found in both the control cell line (lane J, Figure 2) and all tumor extracts. Little of either molecular weight species are seen in any of the pellets, except for a trace of the 73 kDa species in the TD1 pellet. These high apparent recoveries, independent of extraction buffer used, may be due to the hyperglycosylation of this protein, particularly present between the transmembrane helixes. LAMP-3 is found in all the extracts but appears to be in low total abundance overall in the tumor samples because long chemiluminescent substrate exposure times were required to visualize LAMP-3 compared to the other biomarkers tested. Reblotting with higher primary antibody titers did not appear to improve the signal strength (data not shown).

BCL2 is a single-pass apoptosis regulator predominantly found in the outer mitochondrial membrane, but also seen in the nuclear, and endoplasmic reticulum membranes. Recombinant human BCL2 (the first 218 amino acids and nonglycosylated) is used as the antibody control. This truncated recombinant form runs at 24 kDa. Native BCL2 from the tumor samples is seen at 30 kDa [49]. BCL2 recovery was good, but not complete, in all buffers tested, with the best apparent recovery in HEPES buffer. There was no apparent difference in recoveries between the TD1 and TD2 buffers. BCL2 is found in mitochondria. However, because mitochondria are not expected to pellet at the 13,000 ×g used in this experiment [33], it is possible that BCL2 seen in the HEPES extract is actually recovered from intact suspended mitochondria after heating in SDS-PAGE sample buffer. The same may be true of the other buffers, making it difficult to differentiate BCL2 recovery among the Barocycler extraction buffers.

In our work, two CCNE2 bands are seen in the western blot of the cell line control and tumor samples (Figure 2), a dominant band at 45 kDa and a minor band at 47 kDa. These bands probably correspond to the long and short isoforms. As CCNE2 is not a membrane-bound protein, we would expect recoveries to be good in all the buffer systems tested. However, some of the dominant 45 kDa protein remains in the HEPES pellet (lane C). A trace amount is also seen in the TD1 pellet (lane E, Figure 2). These bands might be explained from residual extract present in the unwashed pellets. Extraction appears to be nearly quantitative with the TD2 buffer (lanes F and G, Figure 2). These results could also suggest that one of the components of the TD1 and TD2 buffers may be assisting Barocycler disruption of the nuclear membrane.

When the western blot from our work is probed with a C-terminal-specific GPC3 antibody (i.e., raised against amino acids 303–464) [52], a single strong band is seen in the gel at 30 kDa for all the tumor and cell line samples. The GPC3 protein is lipid anchored to the cell membrane in this C-terminal region. Several splice, or posttranslationally edited, variants are noted in the literature. None of these proteins is extracted into HEPES buffer (lane B, Figure 2). A small amount (*≈*10%) appears to be extracted into TD1 in the sample shown (lanes D and E, Figure 2). About 75% appears to be extracted into TD2 buffer (lanes F and G, Figure 2). This 30 kDa protein is also seen in the cell line control. No other bands are seen in the blot probed with the C-terminal-specific antibody.

When the blot is stripped and reprobed with an antibody specific to the N-terminal GPC3 region [53], however, a single band is seen near 80 kDa in the cell line control (lane J, Figure 2). The 30 kDa fragment is not detected with the N-terminal-specific primary antibody. No corresponding bands in the 60 to 80 kDa region are seen in the gel. Since the mature protein is exported, it would normally be carried away from the tumor site in the blood; therefore, little mature protein is expected to remain in the solid tumor. These results suggest that the 30 kDa fragment may be the lipid-anchored C-terminus (postmodification), which is only recovered in the TD1 and TD2 buffers and that the 80 kDa band is the full-length protein with its lipid-anchored C-terminus intact.

AKT is seen at 60 kDa in the western blot, as determined from the recombinant control (lane I, Figure 2). The second band seen in the recombinant human AKT control sample (at 62 kDa, lane I, Figure 2) is attributed to incomplete cleavage of the His_6_ tag used in the purification of this fusion protein. AKT recoveries are high in all the buffers tested, which would be expected for a soluble cytosolic protein, with no apparent differences between the buffers tested.

While TUBB is generally a cytosolic protein, it spontaneously forms dimers with alpha-tubulin (TUBA) and is always in dynamic equilibrium between soluble a/b-dimers and polymerized microtubules, which can be insoluble depending on their size [57]. A single strong band at 50 kDa is seen in all lanes of the TUBB western blot (Figure 2). Based on the relative chemiluminescent intensities, about 50% of the TUBB is recovered in the HEPES extract (comparison of lanes B and C, Figure 2). TD1 appears to extract more than 80% of the TUBB present in the sample (comparison of lanes D and E, Figure 2). TD2 extracts better than 90% of all the TUBB present. In separate time course experiments (data not shown), we have shown that purified bovine tubulin (Cytoskeleton, Denver, CO) remains soluble at 1 mg/mL in TD1 buffer but polymerizes and precipitates nearly quantitatively within 24 hours in 20 mM HEPES buffer. Therefore, we believe that the lower apparent recovery of TUBB in the HEPES buffer is due to microtubule formation and precipitation in this extract.

### 3.3. Impact of Barocycler and Cryogenic Grinding

Several initial attempts to process diced (unground) metastatic ovarian tumor tissue through the Barocycler with the same extraction buffers did not produce good yields, even for cytosolic proteins (data not shown). Therefore, cryogenic grinding prior to Barocycler extraction appears to be necessary. We speculate that the higher surface-to-volume ratio of the ground tissue allows for better access of the extraction buffer and shorter diffusional paths for the extracted proteins during pressure cycling. Furthermore, as cited above, results with the TD1 buffer proved inconsistent from sample to sample. Some samples yielded small friable pellets and had good protein yields, and others appeared to leave a large sticky pellet after pressure cycling. These latter samples exhibited poor protein recovery. The addition of a dispersion aid (TD2 buffer) resulted in vastly improved sample-to-sample reproducibility. Furthermore, no appreciable protein could be extracted from ground tumor samples heated at 95°C for 1 h in either the TD1 or TD2 buffers in the absence of pressure cycling. When SDS was added to these samples, in the form of SDS-PAGE sample buffer (Invitrogen), protein was subsequently recovered on additional heating (data not shown). This demonstrates that pressure cycling is an integral part of the membrane protein extraction process with the ProteoSolve-TD buffers and that the buffers alone do not act as detergents.

### 3.4. Enzyme-Linked Immunosorbent Assays

**Figure 3:**
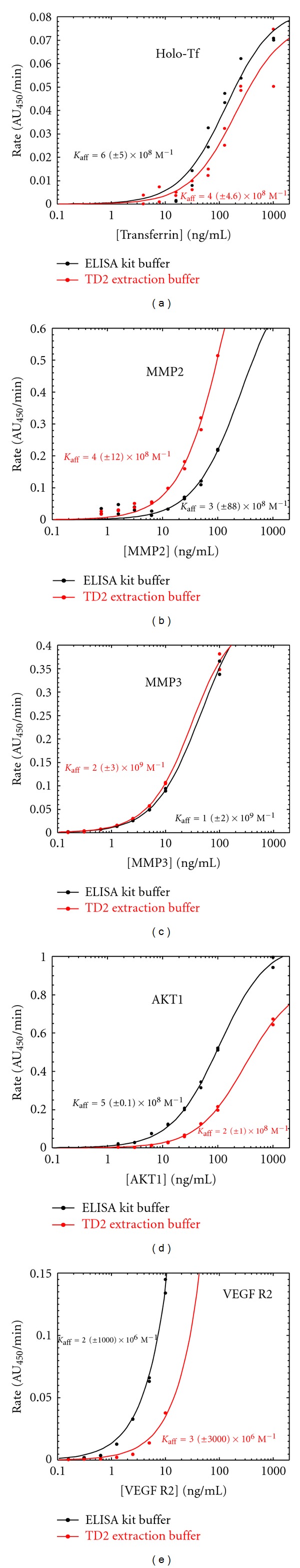
No significant effect of the TD2 buffer was seen in either the rate of color development from the HRP conjugate or antigen affinity in commercial ELISA kits for (a) total human transferrin (Tf), (b and c) total human matrix metalloprotease 2 (MMP2) or 3 (MMP3), (d) total human V-akt murine thymoma viral oncogene homolog 1 (AKT1), or (e) soluble vascular endothelial growth factor receptor (sVEGFR). Apparent affinity constants (±one standard deviation) for separate serial dilutions for the kit standards are shown for each ELISA for both the recommended kit diluent and TD2 buffers. (Standard deviations are determined by partitioning the error of the estimate across the fitted parameters using the Jacobian matrix. In ELISA assays lacking experimental data defining the upper asymptotic limits of quantitation (e.g., VEGF R2 assay of Figure 3), this method produces very large errors when fitting the data with a nonlinear equation (1)).

Several commercial sandwich ELISA kits (transferrin, MMP2, MMP3, AKT1, VEGF, and sVEGF R2) were used to determine the effect of the TD2 extraction buffer on subsequent immunoaffinity work. In each case, one vial of antigen standards was reconstituted as prescribed in the manufacturer's instructions, and a second vial was reconstituted using the TD2 buffer diluted 1 : 10 by volume in ProteoSolve-TDilute (at pH 7.5). Otherwise, the kits were run as prescribed by the manufacturer with the exception that rate assays were performed to determine any residual effects of the TD2 buffer on the reporter (horseradish peroxidase) activity or substrate color development. The resulting standard curves are presented in Figure 3. The small differences in affinity constants between the buffers used are within the experimental error of the serial dilutions and the curve fitting. Furthermore, there was no consistent trend with *K*_*aff*⁡_ being either slightly higher or lower, depending on the assay, and generally within the expected preparation variation of the standards. The lone exception was the VEGF assay, for which no assay response is seen for the recombinant human VEGF_165_ standard reconstituted in the diluted TD2 buffer. Either this standard is not soluble or stable in the TD2 buffer, or the buffer induces a change in the epitope recognized by the ELISA antibodies. Other than the anomalous VEGF assay results, these data suggest that the TD2 extraction buffer has no significant affect on antibody affinity or antigenicity of the recovered proteins. Presumably the kit buffers had been optimized by the manufacturers for each assay. The TD2 extraction buffer was used without optimization in all the assays. We expect, therefore, that better results may be obtained in each assay with further diluent optimization.

The resultant antigen concentrations in the metastatic ovarian tumor TD2 extracts were also determined in these assays and are summarized in Table 3. Holotransferrin (holo-Tf) is a surrogate marker for the blood or serum content of the tumor sample. Serum Tf concentrations are reported to be in the range of 2.9–4.0 × 10^6^ ng/mL [58, 59]. Assuming all of the serum Tf is recovered in the TD2 extract, we estimate 0.5 × 10^6^ ng Tf per g of tissue, suggesting that the average blood/serum content of the frozen metastatic tumor samples is around 15%. Matrix metalloproteinase 2 (MMP2) is below the detection limits of the ELISA (i.e., <70 ng per g of tissue). Matrix metalloproteinase 3 (MMP3) is detectable above background at the highest extract concentration used, but is below the quantitation limits of the assay (i.e., ≤7 ng per g of tissue. No references to the tumor tissue concentrations of these proteins could be found, so recovery could not be determined. AKT1 is a cytosolic protein for which the western blots (Figure 2) suggest nearly quantitative recovery with the TD2 buffer. The total AKT1 concentrations were determined to be about 20 *μ*g/g of tissue, determined from the single ELISA sample tested. VEGF R2 (also known as KDR) is a heavily glycosylated membrane protein. The glycosylated N-terminal domain, which is often cleaved, becomes a plasma-soluble biomarker species (sVEGF R2). Assuming that the antibodies towards sVEGF R2 will crossreact with the membrane bound version, we thought this ELISA kit might provide a more quantitative measurement of the amount of the membrane protein recovered than the western blot (Figure 2), which suggested high recovery efficiency in the TD2 extraction buffer. Polanski and Anderson report the plasma concentration of sVEGF R2 to be 15 ng/mL [59]. The measured tissue concentration of VEGF R2 is 1 ± 0.1 ng/g of tissue, which is about twice as high as that expected for sVEGF R2 in the 15% blood contamination of the tissue sample. This translates to an expected sVEGF R2 concentration of 0.5 ng/g of tissue. However, the amount of sVEGF R2 measured for the HEPES Barocycler extract was indistinguishable from that seen in the TD2 Barocycler extract. Yet, KDR (VEGF R2) recovery in HEPES buffer was poor compared to that observed in the TD2 extraction buffer (Figure 2). Possible explanations are that the ELISA assay is truly specific to the soluble form of this protein, VEGF R2 recovery differs negligibly between the different extraction buffers (i.e., the western blot results are not quantitative), or the amount of membrane-bound VEGF R2 is low relative to that of the sVEGF R2 in the patients' plasma.

### 3.5. Immunoaffinity Enrichment/MALDI-MS

**Figure 4:**
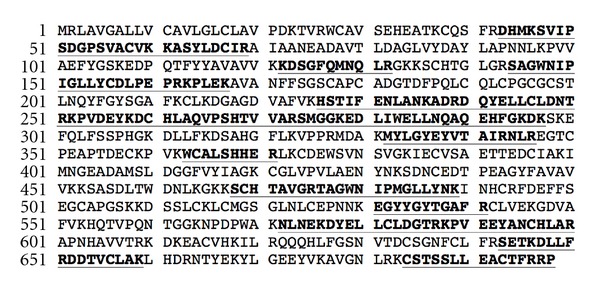
The 36% mass spectrometric sequence coverage (underlined) from a tryptic digest of immunoaffinity-enriched transferrin contained in a Barocycler TD2 extract of metastatic ovarian tumor tissue.

Antitransferrin PhyTips were prepared as described in the methods. These were used to recover and enrich transferrin protein from the TD2 ovarian tumor extract. ELISA data indicate that the extract contains 80 *μ*g of transferrin (Table 3). Digest controls prepared with different concentrations of purified buffer-free apotransferrin (Sigma-Aldrich) suggested a limit of detection of about 4 ng of transferrin in a single MALDI-MS spot (1 *μ*L of sample matrix) for protein identification using peptide mapping. No improvement in sequence coverage was seen above 1 *μ*g of apotransferrin in a 1 *μ*L spot. Of the 34 peaks found in the spectrum and submitted to a MASCOT search, 24 mapped to peptides from human transferrin, which was the top-ranked protein (score of 280). Sequence coverage was 36% (Figure 4).

## 4. Conclusions

### 4.1. Global Protein Recovery and Analysis

Global proteomic comparison between the HEPES and TD2 extracts (Figure 1) shows few differences from among the higher-abundance proteins recovered from the sample. Some 2,000 different proteins have titers greater than 5 × 10^4^ copies in the average mammalian cell [60]. The cellular titers of many cellular receptor (membrane) proteins are reported to be in the range of 10^3^–10^5^ copies per cell, by comparison [61]. The same dynamic range issues that plague global analysis of the plasma proteome [62] also plague cellular proteomic analysis. With the limited dynamic range for a Coomassie-stained gel [63], we may not see lower-abundance membrane proteins in such a global proteomic analysis. Nonetheless, the 2D gel data are important in that they show virtually that all the same proteins are recovered in the TD2 buffer as are recovered in a more standard aqueous buffer and in similar abundance. Furthermore, the ProteoSolve-TD buffers do not affect either the isoelectric focusing or SDS-PAGE separation coordinates of any proteins. Therefore, we conclude that the ProteoSolve-TD buffers are fully compatible with this time-honored global proteomic technique.

### 4.2. Membrane Protein Recovery

The western blots (Figure 2) provide definitive evidence for the recovery of seven-different integral membrane proteins. EDG2 and EDG4, both seven transmembrane G-protein-coupled receptors, appeared to be recovered well in the TD2 buffer system and virtually not at all in the HEPES control buffer. EDG4 recovery, however, may not have been quantitative, but this is confounded by apparent cross-reactivity of the primary antibody with proteins of similar size. FASLG, NRP1, and KDR (VEGF R2) proteins, all single transmembrane proteins, were nearly quantitatively recovered in the TD2 buffer but also showed partial recovery in the HEPES control buffer. LAMP-3, an apparently low-abundance protein with four-transmembrane sequences, appeared to be recovered in good yield in all the extraction buffers. LAMP-3 is highly glycosylated (particularly between the transmembrane helixes), potentially improving its aqueous solubility. Neither overnight incubation or boiling of the tissue samples in the ProteoSolve-TD buffers showed any significant transmembrane protein recovery (data not shown), suggesting that extraction of the membrane proteins was primarily due to the pressure cycling process. These observations support a PCT mechanism (a mechanistic discussion can be found in the Supporting Information) in which the pressure cycle itself is primarily responsible for disruption of the lipid membranes and exclusion of the membrane proteins. The data further suggest that the ProteoSolve-TD buffers need only to support the solubility of the pressure-extracted membrane proteins when the sample is returned to ambient conditions.

GPC3 is a lipid-anchored protein. What appears to be a 30 kDa C-terminal domain, which contains the lipid-anchor, only appears to be recovered in the TD1 and TD2 buffers (Figure 2). The soluble 65 kDa mature GPC3 protein, resulting from cleavage of the C-terminal, lipid-anchored domain, was not seen in any of the samples. BCL2, a mitochondrial protein with a single-transmembrane sequence, was the only protein to show better recovery in the HEPES buffer than either TD1 or TD2 buffers. We believe that this was caused by the failure to pellet mitochondria during the centrifugation step (13,000 ×g). Centrifugation at 52,000 ×g is normally required to pellet free mitochondria [33]. If mitochondria were left suspended in the extracts, then BCL2 would have been liberated from the membranes upon sample preparation for SDS-PAGE (i.e., boiling in SDS sample buffer). Higher recoveries of TUBB were evident in both the TD1 and TD2 buffers compared to the HEPES control. We believe this result is due to improved solubility of tubulin microtubules in the TD buffers over that in HEPES control. By contrast, the soluble protein controls (i.e., CCNE2, AKT, and TUBB) appeared to be well recovered in all of the Barocycler buffers. 

### 4.3. Compatibility with Immunoaffinity Techniques

Of particular clinical interest is that the TD extraction buffers appear to stabilize membrane proteins in an aqueous environment that is compatible with subsequent immunoaffinity techniques (e.g., immunosorbent assays or immunoaffinity enrichment). With performance data from five different ELISAs (Figure 3), we can say with good confidence that the TD buffer system can have negligible effect on antibody affinity constants. Nor does the TD buffer system affect subsequent activity of the final ELISA amplification reaction (at least with the commonly used HRP enzyme). Only a single assay (VEGF) failed with the TD2 extraction buffer system. We believe this may be due to a structural difference in the VEGF epitope in the ProteoSolve-TD buffer system. This might be overcome by the selection of alternative capture or reporter antibodies for the ELISA, but was untried.

Unlike the western blot data (Figure 2), we found no significant difference between sVEGF R2/VEGF R2 titers (by ELISA) between the HEPES and TD2 extraction buffer systems (Table 2). While membrane-bound VEGF R2 (KDR) can be distinguished from sVEGF R2 in a western blot (based on molecular weight differences), these can only be distinguished in an ELISA based on the specificity of the antibodies, which are unknown in the kit used. This ELISA was designed for sVEGF R2 (a plasma marker) and may not be cross-reactive with the membrane-bound version. However, the VEGF R2/sVEGF R2 tissue titers determined (1 ± 0.1 ng/g of tissue) were double the sVEGF R2 titer expected to be present in the entrained blood in the sample [59]. AKT1 (a soluble cytoplasmic protein) was found to be present in the patients' samples at a titer of 20 *μ*g/g of tissue.

We note that both the MMP2 and MMP3 were below the limits of detection or quantitation of the ELISAs used in this study. It seems likely that many cellular proteins of clinical relevance may be similarly too dilute for direct measurement in the small (≤0.2 mL) well volumes of standard ELISA microwell plates. Therefore, we investigated the use of immunoaffinity enrichment to concentrate lower-abundance biomarkers from larger sample volumes for subsequent analysis. Subsequent mass spectrometric analysis was successfully used to confirm the identity of immunoaffinity-enriched transferrin (Figure 4). Not only does this enrichment experiment demonstrate the affinity and avidity of the TUBB antibody in TD2 buffer, but it also shows that none of the buffer components survive the enrichment process to interfere with enzymatic digestion of the sample, peptide ionization (e.g., ion suppression), or mass spectral analysis (e.g., adduct formation).

### 4.4. Compatibility with Mass Spectrometry

Immunoaffinity enrichment followed by direct mass spectrometric determination of the mass of the intact protein to identify possible clinically relevant isoforms was pioneered by Nelson et al. [64] and has been adapted in our laboratory for biomarker validation. While we illustrate this method with a single protein (Tf) to illustrate the method in this paper, however, we have applied it to enrich 33 different biomarkers from the same ovarian tumor samples (data not shown).

As mentioned previously, the TD1 and TD2 buffers are fully compatible with the common protein labeling chemistries (data not provided). We have applied the described immunoaffinity/MS method using isotope-differentiated binding energy shift tags in our laboratory (data not shown) [65]. This intact protein capture approach allows the detection of novel protein isoforms (either sequence variants or posttranslational modifications) that may be lost in other biomarker validation methods such as multireaction monitoring (MRM) [66] or the use of stable isotope standards with antipeptide antibody enrichment (SISCAPA) [67].

Many solid tumors consist primarily of compact epithelial cells and connective tissue, which can be particularly recalcitrant to protein extraction. We note that the solid metastatic ovarian tumors used in this study had to be cryogenically ground to a fine powder before protein extraction proved effective. A video of the sample preparation process is provided in the Supplementary Materials. We also note that the results were variable with the TD1 buffer, but with the addition of the dispersion they aid to create the TD2 buffer, and this recovery variability was eliminated. We suspect that other softer tissues (e.g., liver) or harvested cell lines may be processed via pressure cycling technology with either the TD1 or TD2 buffers and likely will not require prior cryogenic grinding. Pressure cycling technology combined with the commercial ProtoSolve-TD extraction buffers appears to offer a new approach for protein, particularly membrane protein, extraction from tissues in a format suitable for subsequent clinical immunoaffinity methods and classic proteomic analyses.

## Figures and Tables

**Table 1:** Affinity-purified polyclonal antibodies used for western blots and their sources.

Biomarker	Source	Ab Catalog no.	Control cell lysate
Lysophosphatidic acid receptor 2 (EDG4)	Santa Cruz Biotechnology	sc25490	BT-20
Lysophosphatidic acid receptor 1 (EDG2)	Abcam	ab23698	A549
CD95L, tumor necrosis factor ligand (FASLG)	Cell Signaling	4233S	MDA-MB-231
CD304, neurophilin-1 (NRP1)	Santa Cruz Biotechnology	sc7329	MDA-MB-231
CD309, vascular endothelial growth factor receptor 2 (KDR)	Santa Cruz Biotechnology	sc48161	MOLT-4
CD63, lysosomal-associated membrane protein 3 (LAMP-3)	Santa Cruz Biotechnology	sc15363	MOLT-4
Apoptosis regulator 2 (BCL2)	Cell Signaling	2872	MOLT-4
Cyclin-E2, G1/S-specific (CCNE2)	Strategic Diagnostics	2901.00.02	MOLT-4
Glypican-3 (GPC3)	Santa Cruz Biotechnology	sc11395 (30 kDa) sc10455 (60–80 kDa)	MDA-MB-231
RAC serine/threonine-protein kinases (pan-AKT)	R&D Systems	AF2055	MOLT-4
*β*-tubulin (TUBB)	Santa Cruz Biotechnology	sc9935	NIH/3T3

**Table 2:** Salient details of the proteins studied by western blots (Figure 2) and their estimated recoveries following the new method using ProteoSolve-TD2 buffer.

Database accession number	Protein name	Number of transmembrane sequences	Gene name(s)	Predicted sequence MW(s)	Apparent MW(s)	Estimated recovery (ProteoSolve-TD2)
Q9HBW0	Lysophosphatidic acid receptor 2	7	LPAR2 EDG4 LPA2	39 kDa	50 kDa [43]	65%

Q92633	Lysophosphatidic acid receptor 1	7	LPAR1 EDG2	41 kDa	43 kDa [44]	90%

Q0VHD7	CD95 ligand	1	FASLG	31 kDa	40 kDa [45]	95%

O14786	Neurophilin-1 (CD304)	1 0	NRP1 VEGF165R	103 kDa 72 kDa	130–140 kDa [46]	95%

P35968	Vascular endothelial growth factor receptor 2 (CD309)	1	KDR FLK1	150 kDa	150 kDa 200 kDa 230 kDa [47]	90%

P08962	Lysosomal-associated membrane protein 3 (CD63)	4	Tspan-30 LAMP-3	26 kDa	53 kDa [48]	100%

P10415	Apoptosis regulator Bcl-2	1 0	BCL2	26 kDa 22 kDa	28 kDa [49]	see text

O96020	Cyclin-E2 (G1/S specific)	0	CCNE2	47 kDa 41 kDa	45 kDa [50]	100%

P51654 Q53H15 Q2L882 Q2L880	Glypican-3	Lipid anchored 0	GPC3	66 kDa <66 kDa	65 kDa [51–53]	60%

P31749 P3175 Q9Y243	RAC serine/threonine-protein kinases *α*, *β*, *γ*	0	AKT PKB RAC	56 kDa 54 kDa	58 kDa [54]	95%

Q6P602 Q9H4B7 Q13885 Q9BVA1 Q9BUF5 P68371 Q13509 Q2NKY5 Q3ZCM7	*β*-tubulin	0	TUBB [55]	50 kDa	50 kDa [56]	95%

**Table 3:** The concentrations of various protein biomarkers extracted from mixed metastatic ovarian tumors obtained from the omenta of various patients during surgical debulking as measured by ELISA. Proteins were extracted from the cryogenically ground tumor samples using a Barocycler with TD2 Buffer. The concentrations (± one standard deviation) of each biomarker are determined from the extract contained using the ELISA assays described in Figure 3 and extrapolated to that present in the tumor assuming 100% recovery. Transferrin was used as a ubiquitous control protein, which is indicative of the serum content of the sample. Some biomarkers were below the detection limits of the assay. MMP3 was below the limits of quantitation of the ELISA.

Biomarker protein	Extract concentration (ng/mL)	Tumor concentration (ng/g)
Holo-Tf	80,000 ± 40,000	5 × 10^6^ ± 3 × 10^6^
MMP2	BDL (<10)	BDL (<70)
MMP3^∗^	*≈*1	*≈*7
AKT1	40 (single determinant)	20 × 10^3^
VEGF R2/sVEGF R2	0.2 (only 2 replicates)	1 ± 0.1

BDL: below detection limits (assay detection limits).

^∗^Measurements were below the quantitation limits of the assay.
